# The adult outcome of children referred for autism: typology and prediction from childhood

**DOI:** 10.1111/jcpp.13180

**Published:** 2020-01-19

**Authors:** Andrew Pickles, James B. McCauley, Lauren A. Pepa, Marisela Huerta, Catherine Lord

**Affiliations:** ^1^ Department of Biostatistics and Health Informatics Institute of Psychiatry, Psychology & Neuroscience King’s College London London UK; ^2^ Department of Psychiatry University of California, Los Angeles Los Angeles CA USA; ^3^ Centre for Autism and the Developing Brain Weill Cornell Medicine New York NY USA; ^4^ Felicity House New York NY USA

**Keywords:** Childhood, autism spectrum disorders, adult outcome, prediction, EDX

## Abstract

**Background:**

Autism Spectrum Disorder is highly heterogeneous, no more so than in the complex world of adult life. Being able to summarize that complexity and have some notion of the confidence with which we could predict outcome from childhood would be helpful for clinical practice and planning.

**Methods:**

Latent class profile analysis is applied to data from 123 participants from the Early Diagnosis Study (Lord et al., Archives of General Psychiatry, 2006, 63, 694) to summarize in a typology the multifacetted early adult outcome of children referred for autism around age 2. The form of the classes and their predictability from childhood is described.

**Results:**

Defined over 15 measures, the adult outcomes were reduced to four latent classes, accounting for much of the variation in cognitive and functional measures but little in the affective measures. The classes could be well and progressively more accurately predicted from childhood IQ and symptom severity measurement taken at age 2 years to age 9 years. Removing verbal and nonverbal IQ and autism symptom severity measurement from the profile of adult measures did not change the number of the latent classes; however, there was some change in the class composition and they were more difficult to predict.

**Conclusions:**

While an empirical summary of adult outcome is possible, careful consideration needs to be given to the aspects that should be given priority. An outcome typology that gives weight to cognitive outcomes is well predicted from corresponding measures taken in childhood, even after account for prediction bias from fitting a complex model to a small sample. However, subjective well‐being and affective aspects of adult outcome were weakly related to functional outcomes and poorly predicted from childhood.

## Introduction

A rare consensus in the field of autism spectrum disorder (ASD) is the heterogeneity (Billstedt et al., 2005; Bishop‐Fitzpatrick et al., 2016; Howlin, Mawhood, & Rutter, 2000; Magiati, Tay, & Howlin, 2014; Seltzer, Shattuck, Abbeduto, & Greenberg, 2004). Previous diagnostic divisions such as Asperger’s syndrome and PDD‐NOS have given way to a spectrum of ASD. This implies that there may be considerable variation in severity in terms of symptomatic behaviours that may be measured by such scores as the Comparative Severity Score on the Autism Diagnostic Observation Schedule (ADOS; Gotham, Pickles, & Lord, 2009). These scores were not, however, constructed to provide a global or even partial assessment of an autistic individual’s capacities. By adulthood, a period far longer than childhood, a rounded description of outcome involves consideration of a diverse range of domains within which measures of autism symptomatology will be just one of many (Jones et al., 2018; Mazefsky, Folstein, & Lainhart, 2008). Such a diversity of measures offers many potential dimensions of variability, substantially confusing the task of prioritizing targets and the planning that patients, parents and clinicians must all face. Summarizing heterogeneity is essential for clarity of communication and, arguably, for decision‐making and action, but this risks oversimplification. This is a particular concern when considering the multifacetted needs and demands made on adults, when the sources of support are more fragmented than in childhood (Howlin & Moss, 2012; Shattuck, Wagner, Narendorf, Sterzing, & Hensley, 2011). Some previous work side‐stepped this problem by examining the small minority whose adult outcomes were good across multiple domains (e.g. Fein et al., 2013). Succinctly characterizing adult outcome across the whole range is a harder task.

We make use of the Autism Early Diagnosis Cohort (EDX; Anderson et al., 2007; Lord et al., 2006) of children referred for possible autism. In a previous paper Lord, McCauley, Pepa, Huerta, and Pickles (2019) used three outcome groupings identified *apriori,* based on ASD clinical history and adult IQ (Never ASD, ASD‐High IQ, ASD‐Low IQ). They then compared these groups over a battery of measures chosen to reflect a broad range of adult functioning, including adaptive skills, vocational activities and behavioural and emotional problems. Here, we examine the same set of adult measures, to see whether the adult profiles themselves form groupings of more frequently occurring ‘types’, how many types provide an effective summary, and what is distinctive about each one. We describe which aspects of adult life vary together and are thus better explained by this typology, as well as considering other aspects that follow these more central patterns more loosely or very little at all. We then examine whether measures, fairly typical of those made in current clinical assessments and taken when the children first entered the cohort, are predictive of their adult outcome group, and the precision of prediction that can be achieved. Finally, we examine how that prediction improves as the child develops from 2 to 9 years‐of‐age, to identify at what age a more confident prognosis could have been made for this cohort. Wanting to mirror the prospective challenge of the clinician considering the adult outcome of a child whose future ASD diagnosis may or may not be consistent with their current presentation, we included all children, including the small minority who, as it turned out, never met the clinicians’ criteria for ASD.

## Methods

### Participants

The participants used to characterize the adult outcome classes were drawn from three sources: (a) 192 children under age 3 years who were referred for possible autism to two tertiary autism programs (North Carolina and Chicago), (b) 21 children of the same age and sources recruited as a comparison group that had never been referred to the autism programs and who, though exhibiting developmental delay, were not thought to have ASD at that time and (c) 31 new recruits from Michigan who joined the study at approximately age 9 and recruited from sources similar to those used at project inception (see Anderson, Liang, & Lord, 2014). The latter group were not included in the predictive analyses.

Face‐to‐face assessments were undertaken for all children and parents who could be reached at age 2, 3 (ASD referrals only), 5 (North Carolina only), 9, 19 and 25 years‐of‐age, with some young adults seen for an additional assessment at 21. Of the original 213 participants recruited at age 2, losses occurred due to geographical relocation and unreachable status (the largest loss was between ages 5 and 9 when cell phones came into common use) with just 24 (11.3%) refusing ongoing participation. Overall attrition of 61.5% was higher in African‐American families and families with the lowest educational levels. No effects of gender, earlier diagnoses or IQ on attrition have been found at any point. This study includes 106 young adults from the EDX study who participated in at least two assessments, at least one of which was between age 22 and 27. Of the 31 recruited later, 17 were seen on at least one additional occasion as an adult and provided sufficient data to be included. This group differs significantly from the original sample in that there are more females and more Caucasians and parental education was lower. We controlled for attrition‐associated factors in all analyses.

For the analysed sample, the mean ages for joining the study varied for those initially sampled (*M* = 2.5 years, *SD* = 0.43) and the new recruits (*M* = 8.56 years, *SD* = 2.85). Mean ages for most recent assessments used were 26.15 years (*SD* = 1.47) and 25.00 years (*SD* = 1.84), respectively. Ethnic minorities, almost all of whom were African American, accounted for 16% of the mostly male (85%) sample, with a mix of families originally from rural, suburban and urban backgrounds (61 from North Carolina, 45 from Chicago and 17 from Michigan). Mothers were mostly married at the time of the child’s birth (92.5%) and had college degrees (62%). Review of all assessments diagnosed 75% as ASD (Autism and PDD‐NOS) at the age 2 contact, with a further 3% receiving new ASD diagnoses at age 5 and a further 7% at age 9.

### Procedures

A battery of diagnostic and psychometric instruments was administered in person during home, school, work and clinic visits, arranged with families and participants at their convenience. As much as possible, clinicians administered test batteries blind to results from previous assessments, including diagnosis, though often families talked about earlier diagnoses once the assessment began. In general, assessments were carried out by a team of one Ph.D. level psychologist, an advanced graduate student and/or one or two postbaccalaureate research assistants who had achieved research reliability on the measures they administered. Informed consent was obtained from all participating families and, whenever possible, individuals themselves. The research was approved by the Weill Cornell Medicine IRB as well as earlier IRBs at Universities of Michigan, Chicago and North Carolina.

### Measures

These are described more fully in Appendix S1 in the Supporting Information but included the ADI‐R (Lord, Rutter & Le Couteur, 1994) and the comparative severity score (CSS) from the Autism Diagnostic Observation Schedule (ADOS; Gotham et al., 2009; Lord et al., 2000, 2012); Verbal and nonverbal IQs (VIQ, NVIQ) from Wechsler Abbreviated Scale of Intelligence (Wechsler, 1999), Differential Ability Scales (Elliott, 2007) and the Mullen Scales of Early Learning (Mullen, 1995); the Social Emotional Functioning Interview (Howlin et al., 2000; Rutter et al., 1988); the Well‐Being Questionnaire (WBQ, Ryff, 1989); the Beck Depression Inventory (BDI‐II; Beck, Steer, & Brown, 1996); the two‐subscale scores from the Positive and Negative Affect Schedule (PANAS‐P and PANAS‐N; Watson, Clark, & Tellegen, 1988); the Daily Living standard score from the Vineland Adaptive Behavior Scales (Vineland II; Sparrow, Cicchetti, & Balla, 2005); the total problem score from the Adult Behavior Checklist (ABCL; Achenbach & Rescorla, 2003); irritability, hyperactivity and medication use (drug and dose) from the Aberrant Behavior Checklist (Aman, Singh, Stewart, & Field, 1985). Missing items in item‐totals were prorated where 80% items were otherwise complete.

### Statistical analysis

Latent profile analysis was undertaken in Stata 15 using the gllamm procedure (Rabe‐Hesketh, Skrondal, & Pickles, 2004). Of the 15 variables over which the profiles were defined, the CSS, Work, Living and Friends scales were treated as ordinal, the number of medicines as overdispersed Poisson, and the remaining ten variables as Gaussian. For ease of analysis and presentation, these latter variables were first standardized and, where required to achieve a consistency of interpretation with all other measures, were reversed to ensure that higher scores implied a poorer outcome. Models were estimated using full‐information maximum likelihood so that participants with partially incomplete profiles were included in the analysis under the missing‐at‐random assumption. Estimated with an incrementing number of classes, the Bayesian information criterion (BIC) was used in the choice of a parsimonious model.

Individuals were assigned to their most likely class for second‐stage analysis. The identified solution generated class assignments with a high level of classification certainty, allowing us to proceed with this second stage directly, rather than using the more complex methods that are required where classification is poorer. For the second‐stage analyses, we used multinomial regression and associated prediction probabilities. It is well known that examining prediction success within the same sample as that used to estimate the model leads to an overoptimistic assessment of predictive performance. We estimated an optimism‐bias correction (Harrell, 2001), obtained as the mean estimate over 100 bootstrap samples of the difference between the performance of the predictions based on our model estimated on the whole sample and the higher estimate obtained from fitting the same prediction model to the bootstrap sample. Bias‐adjusted estimates of performance were obtained by subtracting the optimism bias from the naïve performance estimates.

## Results

Table 1 summarizes the whole sample at the time of recruitment and the 15 variables used to characterize adult outcome. As shown in Table 2, on incrementing the number of classes in the latent class profile model, the BIC decreased from the two‐class model (3,385.57) to the three‐class model (3,265.75) and from there to the four‐class model (3,199.64). However, for the five‐class model the BIC clearly worsened (3,231.98), suggesting that the additional complexity of a fifth class was not justified by sufficient improvement in model fit.

**Table 1:** Summary statistics for the analysed sample

	No. of obs.	Mean/percent	Standard Deviation	Range
At recruitment				
Non‐Caucasian	123	17%		0:1
Maternal education	123	2.24	1.09	1:5
Comparative severity score	120	6.32	3.07	1:10
Verbal IQ	123	45.66	28.44	10:123
Nonverbal IQ	123	71.54	24.19	13:132
Female	123	17%		0:1
Age in years	123	3.29	2.41	1.3:11.83
Adult outcome				
Comp.Sev.Score	118	5.57	2.72	1:10
Verbal IQ	123	60.70	43.81	2:139
Nonverbal IQ	123	64.02	40.49	3:133
Hyperactivity	104	7.58	7.77	0:30.75
Irritability	104	6.50	7.26	0:37.5
ABCL total	94	52.33	8.80	25:77
Beck Depression	92	4.81	6.02	0:30
PANAS Pos.	92	28.46	8.15	12:45.5
PANAS Neg,	93	17.18	6.23	10:35.5
Well‐being WBQ	91	189.16	25.90	134:248
Num of Meds.	99	1.37	1.38	0:5
Work	113	4.05	2.28	1:7
Living	123	2	0.61	1:3
SEF friends	106	1.65	1.21	0:3
Daily living	123	59.36	25.15	17:112

**Table 2:** Latent class model likelihoods and Bayesian information criteria (BIC) for selecting number of classes for profiles over the full and the reduced sets of outcome measures (minimum BIC highlighted)

Number of classes	Number of parameters	Log‐likelihood	BIC for full profile with all measures (IQ reduced profile) [IQ and CSS reduced profile]
2	57	1,555.64	3,385.57 (3,356.76) [2,817.08]
3	73	1,457.23	3,265.75 (3,278.24) [2,732.20]
4^a^	89	1,385.68	**3,199.64 (3,268.01) [2,716.63]**
5	105	1,363.35	3,231.98 (3,284/79) [2,727.24]

Full profile: Class1 22% 0.996, Class2 26% 0.94, Class3 25% 0.96, Class4 27% 0.96;

IQ reduced profile: Class1 18% 0.97, Class2 35% 0.95, Class3 27% 0.95, Class4 20% 0.98

IQ + CSS reduced profile: Class1 0.97 25%, Class2 0.98 41%, Class3 0.90 13%, Class4 0.95 20%.

a Prevalence percentage and mean posterior class probabilities for 4 class solutions.

**Figure 1:**
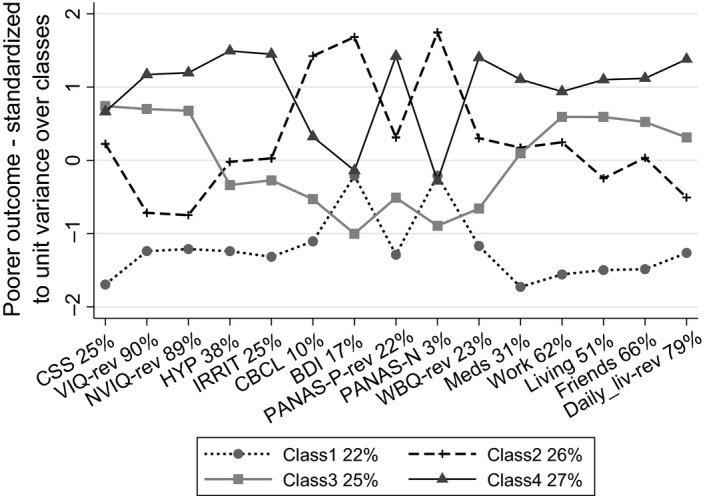
Latent class profiles for the complete set of 15 adult outcome measures. Variable labels identify reversed scales and the percentage of the variance of that variable explained by the latent classes. The legend shows the prevalence of the latent classes as proportions of the original cohort recruits

For the four‐class model, the average class assignment probabilities of those assigned to Class 1 were 0.996, with corresponding probabilities of 0.942, 0.962 and 0.956 for those assigned to classes 2, 3 and 4, respectively. The classes were of surprisingly equal size, with 21% assigned to class 1 and 26% for each of the 3 other classes. Weighted to account for the attrition since initial recruitment, the class prevalences were little changed (22%, 26%, 25% and 27% of the original cohort, respectively). Table 3 gives the summary statistics for each class and Figure 1 displays this graphically, where the continuous scores representing the explained variability have been standardized to zero mean and variance of one (for the sample as a whole) and some scales are labelled as reversed such that higher scores consistently indicate a poorer outcome. Though too small for inferential conclusions, weighted rates of class proportions for the 17 women were 7%, 17%,50% and 28% and for the 17 non‐Caucasians 5%, 34%, 26% and 34%.

**Table 3:** Outcome profiles for participants assigned by class: means (standard deviations) [number of observations]

Measure	Class 1 Best Outcome	Class 2 High‐IQ ASD	Class 3 Low‐IQ ASD without behavioural problems	Class 4 Low‐IQ ASD with behavioural problems
Comp.Sev.Score	3.2 (1.7) [29]	6.0 (2.5) [28]	6.6 (2.2) [31]	6.4 (2.9) [30]
Verbal IQ	112.4 (14.0) [31]	90.1 (20.6) [29]	32.8 (10.7) [31]	11.0 (7.8) [32]
Nonverbal IQ	110.2 (13.5) [31]	92.5 (14.8) [29]	38.7 (15.7) [31]	18.0 (11.0) [32]
Hyperactivity	1.8 (1.9) [24]	7.5 (7.6) [23]	5.7 (5.3) [30]	14.9 (8.0) [27]
Irritability	1.7 (2.4) [24]	6.6 (6.8) [23]	5.4 (5.5) [30]	11.9 (8.8) [27]
ABCL total	49.0 (7.9) [26]	56.5 (10.0) [24]	51.0 (7.8) [23]	53.2 (8.0) [21]
Beck depression	4.4 (5.2) [19]	8.9 (8.6) [21]	2.2 (3.6) [28]	4.5 (4.4) [24]
PANAS Pos.	33.4 (6.9) [19]	27.0 (7.0) [21]	30.6 (7.2) [29]	22.9 (7.9) [23]
PANAS Neg.	17.0 (7.3) [19]	19.1 (6.6) [21]	16.3 (6.0) [29]	16.7 (5.1) [24]
Well‐being WBQ	205.1 (20.6) [19]	183.7 (22.1) [21]	197.0 (25.0) [27]	172.5 (23.8) [24]
Num. of Meds.	0.3 (0.7) [21]	1.3 (1.3) [21]	1.2 (1.3) [30]	2.5 (1.3) [27]
Work	1.3 (0.6) [31]	4.3 (2.2) [28]	5.1 (1.5) [27]	5.9 (1.0) [27]
Living	1.3 (0.5) [31]	2.0 (1.0) [29]	2.2 (0.4) [31]	2.5 (0.5) [32]
SEF friends	0.2 (0.5) [29]	1.7 (1.0) [26]	2.1 (0.8) [31]	2.8 (0.4) [26]
Daily living	87.7 (12.3) [31]	69.7 (12.4) [29]	53.8 (13.6) [31]	27.8 (7.7) [32]

The variable labels in Figure 1 also indicate the simple percentage of the variance of each variable explained by the latent typology. These percentages show how IQ, both verbal and nonverbal, and the functional measures of the Vineland Daily Living Scale, Friendships, Work and Living circumstances are central to the typology, since the differences between the classes explain much of their variability. By contrast, the variation in the parent‐rated behavioural, mental health and well‐being outcomes is, for the most part, not well described by this typology, a possible exception being hyperactivity. Variation in ASD symptoms, as measured by the CSS assessed in adulthood, is also only modestly explained by the typology. Since variation in measures not explained by variation between the latent groupings must be reflected by variation within each of the groups, a corollary is that each class, while relatively homogeneous with respect to ability, skills and circumstance, possesses substantial internal variability in ASD symptoms and cooccurring parent‐rated behavioural, mental health and well‐being.

Class 1, displays the least ASD symptomatology, has high IQs, both verbal and nonverbal, and shows a near uniformly good functional and behavioural outcome. The only exceptions are parent reports of depressive (BDI) and negative emotions in the participants (PANAS‐N). However, examination of the class‐specific raw data means of Table 3 for the BDI and PANAS‐N show, with the exception of BDI for Class 2, only modest class differences, consistent with this particular typology not explaining well the variation of these variables. We refer to this class as the Best Outcome class. Class 2 displays autism symptoms only a little above the average of the sample and IQs only a little below the mean for the general population and a little lower than Class 1. Nonetheless, this class shows a markedly poorer outcome across the span of other measures; functional, behavioural and affect. We refer to this class as the High‐IQ ASD Outcome class. Classes 3 and 4 share the same high level of autistic symptomatology, have the lowest verbal and nonverbal IQs, and the poorest functional outcomes. However, those in Class 4 display higher rates of cooccurring behavioural problems, notably hyperactivity and irritability, more limited daily living skills and reported well‐being and a poor, though inconsistent, profile over the affective domain. We refer to Class 3 as Low IQ ASD Without Behavioural Problems class and Class 4 as the Low IQ ASD With Behavioural Problems class. A comparison with the *apriori* groups of Lord et al. (2019), Class 1 comprised of 25 individuals from their ASD‐High IQ group, and 6 individuals from the Never ASD group; Class 2 included 20 ASD‐High IQ, 5 from ASD‐Low IQ, and 4 Never ASD individuals; Class 3 included 23 ASD‐L group and 8 Never ASD individuals; Class 4 included 31 ASD‐Low IQ, and 1 Never ASD individual.

### Predicting outcome

**Figure 2:**
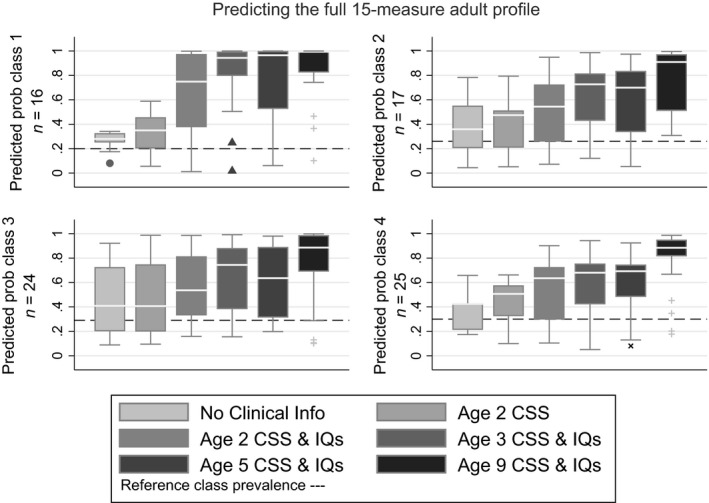
Median and spread of prediction performance for children assigned to each adult‐outcome class: 1 = Best outcome, 2 = High‐IQ ASD, 3 = Low‐IQ ASD without behavioural problems, 4 = Low‐IQ ASD with behavioural problems

Using standard multinomial logistic regression, we next examined our ability to predict class membership using key variables collected over time since first assessment. Our predictive performance is shown in Figure 2. The dashed horizontal line on each chart shows what our chance prediction success would be if we simply assigned children at random based on knowledge of the class prevalence.

#### Prediction from nonclinical baseline

The first box plot in each panel shows the predicted probability for each child assigned to that class, based on the nonclinical variables of the child’s race, gender, parental education and the recruitment site. The extent to which the median line of the boxplot lies above the horizontal line indicates how these nonclinical variables alone influence adult outcome. The highest sections of the boxplot are indicative of how well we predict for some children. The parts of the boxplot falling below the horizontal line highlights the children for whom our predictions are worse than chance. We retained these nonclinical variables in the model, even though nonsignificant as predictors, since these provided a relevant benchmark against which to assess our clinical prediction ability.

#### Prediction from first assessment

The second boxplot of each panel follows the addition as a predictor of the CSS score when first assessed at age 2. While a significant addition (*χ* ^2^(3) = 8.31, *p* = .040), the Figure shows that the increment in predictive performance is quite modest, with the median prediction probability exceeding 0.5 for no class. The third boxplot shows the prediction improvement on adding both verbal (*χ* ^2^(3) = 10.18, *p* = .017) and nonverbal IQs(*χ* ^2^(3) = 8.23, *p* = .041). These all show a rise in the median predicted probability, for some classes substantial. The Vineland Daily Living Skills at age 2 proved nonsignificant as an additional predictor (*χ* ^2^(3) = 0.46, *p* = .927).

The mean naïve prediction probabilities for the model with nonclinical, CSS and IQ measures as predictors were 0.66, 0.54, 0.57 and 0.55 for classes 1, 2, 3 and 4, respectively. Subtracting the bootstrap optimism bias due to examining prediction success in the same sample as that used to estimate the prediction model provided bias‐adjusted estimates of success of 0.58, 0.44, 0.49 and 0.49.

#### Age incrementing prediction

From this point, we took the model with nonclinical, CSS and IQ predictors as the baseline (with the model coefficients fixed) and examined the impact on prediction success of subsequent assessments of CSS, verbal and nonverbal IQ at ages 3, 5 and 9. The fourth, fifth and sixth boxplots in each panel of Figure 2 show that there is a marked improvement in prediction success from age 2 to age 3, little improvement between ages 3 and 5 (the age 5 measures sometimes appearing worse than those at age 3), but by age 9, the naïve prediction probabilities are high, 0.84, 0.77, 0.76 and 0.80. Once corrected for optimism, the bias‐adjusted estimates were 0.80, 0.72, 0.73 and 0.77. Identifying which updated variables deliver this improvement is hazardous on a sample this small, but it can be noted that while the age 9 IQ measures were a significant addition over the age 2 model (6 *df* Wald test *p* = .011), the age 9 CSS was not (3 *df* Wald test *p* = .634).

#### Robustness of prediction to changes in outcome definition

We were concerned that the importance of the IQ measures as predictors of outcome was due to these measures also seeming to cohere strongly as the core of the variability of the adult outcome latent classes – that we were merely seeing child IQ predict adult IQ. We therefore repeated the whole analysis excluding VIQ and NVIQ measures from the profile of adult measures contributing to the adult outcome latent class. The 4‐class model again proved parsimonious giving the profiles of class means shown in Figure S1. The identified classes appear superficially similar to those found previously (Table S1) and again had mean class assignment probabilities that all exceeded 90%, but continued to explain little of the variation in the parent‐rated BDI, PANAS or WBQ measures (Figure S1). However, these new outcome classes were more difficult to predict from our set of childhood measures (Figure S2). After applying bias correction, the figures for prediction for classes 1–4 from age 2 were 34%, 58%, 31% and 23%, and from age 9 were 59%, 63%, 47% and 39%.

Repeated a third time, additionally removing the CSS from the adult profile of measures, the latent class analysis again identified four classes as the most parsimonious. Figure S3 shows that these classes now account for substantial variability in most variables, the exceptions being parent ratings of the positive dimension of the PANAS and of Well‐Being (Table S2). These new outcome classes were still more difficult to predict from our set of childhood measures (Figure S4). After applying bias correction, the figures for prediction for classes 1–4 from age 2 were 32%, 53%, 9% and 27%, and from age 9 were 44%, 58%, 31% and 44%.

## Discussion

This paper has been concerned with the outcomes for a complete cohort of children referred for possible autism, whether we can identify a limited number of types of outcome, and how well we can predict this typology from the kind of data commonly collected during clinical assessments in childhood.

While clear that a typology of adult outcomes can be empirically extracted from a set of measures that can classify with confidence young adults with varying degrees of autism, we caution their interpretation as an aetiological taxonomy. It was evident that our typologies placed greater weight on those measures that covary together, generating groups that were relatively homogeneous with respect to IQ but, particularly in the affective domain, almost as heterogeneous as the whole original cohort. The multifacetted nature of adult life makes the choice of measures to capture its overall quality somewhat arbitrary. The exclusion of verbal and nonverbal IQ from the set of measures had a mixed effect on how the overall outcome groups were partitioned, some classes remaining near unchanged whereas others reflected a mix of individuals whose outcomes had not been grouped together before. The exclusion of these IQ measures, and additionally the CSS, from the adult measures began to provide groupings that had a little more separation in the affective domain.

Despite the limitations in many aspects of intelligence testing, verbal and nonverbal IQ, even at age 2, and more so by age 3, were strong predictors of independence at age 26. It is important to note that predictive IQs at preschool were not high in terms of absolute scores (Table 1); IQ scores for the Best Outcome class were below average during most of the preschool years, but showed steady increases throughout childhood, only reaching above average IQ scores in adulthood. Alvares et al (2020) highlight the imperfect relationship between IQ and contemporaneous functioning as measured by the VABS, especially for those without ID, and that IQ alone is an inadequate basis for defining ‘high‐functioning autism’. Nonetheless, for long‐term prognosis from childhood, IQ seems to be our best predictor even of an outcome typology that excludes IQ. Recent years has seen IQ being given less attention for a variety of reasons (Stedman, Taylor, Erard, Peura, & Siegel, 2018), being omitted from the measurement batteries of several longitudinal studies of adults. Similarly, intervention studies that result in increased IQs without concomitant changes in ASD symptoms have tended to be perhaps somewhat undervalued.

Though significant, autism CSS at age 2 did not contribute to predictions as much as IQ, though it was apparent that the Best Outcome group was quite different from the other groups in their CSS scores on the ADOS in adulthood. Because this is a study of referrals for possible autism, some never received an ASD diagnosis over repeated assessments, and these 12 participants were restricted to the Best Outcome (7) and High‐IQ ASD (5) classes. However, while updating IQ to age 9 improved prediction substantially, updating the CSS to age 9 did not add significantly. That demographic data did not achieve prediction much better than chance may be seen as some evidence against there being gross social inequity in overall outcomes.

Evidence of our ability to predict from early measures was balanced by evidence from the boxplots that, on occasion, individual child outcomes can surprise us, with some children achieving outcomes considered unlikely even as they presented at age 9. Moreover, although we used bias correcting techniques to overcome the risk of exaggerated claims of prediction success, any generalization must be cautious. The cohort is small and fixed in its historical and familial context. The heterogeneity in diagnostic, therapeutic and educational services received over their lifetimes, while recorded in detail, are so varied and mixed that quantifying their impact, and accounting for it, has not been possible. These cautions, and more, must apply especially to females and nonwhite children, whose numbers in this study are very small.

Finally, we have noted how variation in parent ratings of positive affect or well‐being contribute less than anticipated to the definition of the latent typology. One might have expected these ratings, the most overtly related to happiness, to be central, but this does not seem to be the case. It should be noted that in neurotypical populations, while significantly associated, subjective and objective measures also show discrepancies, possibly due to ‘adaptive preference formation’ and ‘lower expectations’ (Henninger & Taylor, 2013; Western & Tomaszewski, 2016) that may be more pronounced in our sample. This would suggest that, alongside improvements in prognostic tools, there is a need for greater clarity as to what the desired outcome of long‐term therapeutic involvement should be – a priority setting task that is perhaps long overdue.

## Supporting information


**Appendix S1** **.** Measures.
**Table S1** **.** Outcome profile classes when leaving out verbal and nonverbal IQ from the profile of measures: means (standard deviations) [number of observations].
**Table S2** **.** Outcome profile classes when leaving out verbal IQ, nonverbal IQ and Comparative Severity Score from the profile of measures: means (standard deviations) [number of observations].
**Figure S1** **.** 4‐class adult outcome latent profile omitting adult verbal and nonverbal IQ.
**Figure S2** **.** Prediction of adult outcome classes for profile omitting adult verbal and nonverbal IQ.
**Figure S3** **.** Classes identified from profiles omitting verbal IQ, nonverbal IQ and Comparative Severity Score.
**Figure S4** **.** Prediction of adult outcome classes for profiles omitting verbal IQ, nonverbal IQ and Comparative Severity Score.
