# Supplementary material for: The adult outcome of children referred for autism: typology and prediction from childhood
Source: J Child Psychol Psychiatry. 2020 Jan 19;61(7):760–7. doi: 10.1111/jcpp.13180 (PMC7384105; doi:10.1111/jcpp.13180)
Supplement: Supplementary file 1 — Appendix S1 . Measures. Table S1 . Outcome profile classes when leaving out verbal and nonverbal IQ from the profile of measures: means (standard deviations) [number of observations]. Table S2 . Outcome profile classes when leaving out verbal IQ, nonverbal IQ and Comparative Severity Score from the profile of measures: means (standard deviations) [number of observations]. Figure S1 . 4‐class adult outcome latent profile omitting adult verbal and nonverbal IQ. Figure S2 . Prediction of adult outcome classes for profile omitting adult verbal and nonverbal IQ. Figure S3 . Classes identified from profiles omitting verbal IQ, nonverbal IQ and Comparative Severity Score. Figure S4 . Prediction of adult outcome classes for profiles omitting verbal IQ, nonverbal IQ and Comparative Severity Score. [file JCPP-61-760-s001.docx]

**Supporting information – The adult outcome of children referred for autism: typology and prediction from childhood – by Pickles *et al*.**

**Appendix S1.** Measures.

The Autism Diagnostic Interview – Revised, an investigator-based interview, was administered to caregivers in both childhood and adulthood. The Autism Diagnostic Observation Schedule (Bal & Lord, 2015; Lord et al., 2000, 2012) is a standardized observation of social communication and play or imaginative use of materials that was administered by a trained examiner. For the adult assessments, typically Module 4 was administered to verbally fluent participants, unless they requested to use the materials in Module 3. The Adapted Autism Diagnostic Observation Schedule (Adapted ADOS) Modules 1 or 2 was administered to participants who were minimally verbal or spoke in simple sentences or single words. The calibrated severity scores (with algorithms from Module 1 and 2 applied to the Adapted ADOS) were used to summarize these measures. On the basis of all available information, including the ADOS and ADI-R, participants were given a current diagnosis of ASD or another disorder or no diagnosis.

Cognitive skills at the age 19 visit (or later if this visit was missed) used the following hierarchy of tests if possible: Wechsler Abbreviated Scale of Intelligence (WASI; Wechsler, 1999), Differential Ability Scales (DAS II; Elliott, 2007) and the Mullen Scales of Early Learning (Mullen, 1995). Ratio IQs were calculated when raw scores fell outside the ranges for deviation scores.

Verbally fluent adults meeting the overall language criteria for an ADOS Module 3 or 4, were also given the Social Emotional Functioning Interview (SEF-I), a series of open-ended questions about social preferences, concerns and interests (Rutter et al., 1988). Caregivers for all participants were given the informant version of the same scale (Howlin et al., 2000). Summary codes for “work”, place of living, and educational attainment used information from caregivers, and whenever possible, from the participants themselves.

Completed by the caregiver, total scores from the Well Being Questionnaire (WBQ, Ryff, 1989), the Beck Depression Inventory (BDI-II; Beck, Steer, & Brown, 1996), and the two-sub-scale scores from the Positive and Negative Affect Schedule (PANAS; Watson, Clark, & Tellegen, 1988) were used in adult assessments. In the case of the BDI and the PANAS, higher numbers indicate the person or caregiver endorsed more items describing depressive symptoms, negative emotions (PANAS-N) or pleasant emotions (PANAS-P). A higher WBQ score indicates more statements about good well-being.

Adaptive skills were assessed using the Vineland Adaptive Behavior Scales (Vineland II; Sparrow, Cicchetti, & Balla, 2005) through an interview with the parent, caregiver or occasionally an informant. Caregivers completed the Aberrant Behavior Checklist (ABC; Aman, Singh, Stewart, & Field, 1985), provided irritability and hyperactivity scores, and the Adult Behavior Checklist (ABCL; Achenbach & Rescorla, 2003) behaviour problem totals. Medication use (drug and dose) was reported on the ABC questionnaire and confirmed with caregivers and participants.


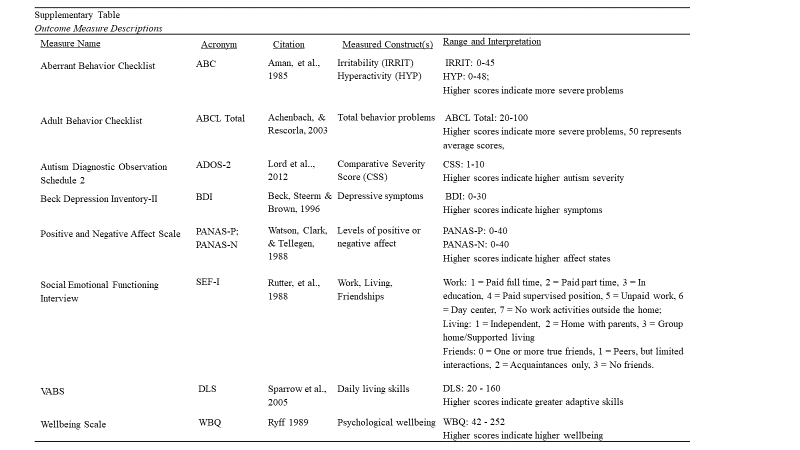


**Figure S1.** 4-class adult outcome latent profile omitting adult verbal and non-verbal IQ.

**Figure S2.** Prediction of adult outcome classes for profile omitting adult verbal and non-verbal IQ.

**Table S1.** Outcome profile classes when leaving out verbal and non-verbal IQ from the profile of measures: means (standard deviations) [number of observations].

| Measure | Class 1  Best outcome | Class 2  Low-IQ ASD with few behavioural and emotional problems | Class 3  Higher-IQ ASD | Class 4  Low-IQ ASD with behavioural and emotional problems |
| --- | --- | --- | --- | --- |
| Comp.Sev.Score | 2.7 (1.4) [20] | 7.1 (2.4) [44] | 5.0 (2.4) [32] | 5.9 (2.3) [22] |
| Verbal IQ | 112.6 (15.1) [22] | 32.9 (30.8) [45] | 86.5 (29.8) [32] | 30.9 (31.8) [24] |
| Non-verbal IQ | 109.5 (14.0) [22] | 37.9 (29.3) [45] | 89.5 (27.1) [32] | 37.3 (30.3) [24] |
| Hyperactivity | 1.4 (1.8) [16] | 6.2 (4.1) [40] | 3.5 (3.4) [26] | 19.5 (6.8) [22] |
| Irritability | 1.5 (2.6) [16] | 4.8 (3.8) [40] | 2.8 (2.6) [26] | 17.6 (6.9) [22] |
| CBCL total | 46.8 (7.4) [17] | 50.1 (8.3) [39] | 55.0 (8.2) [23] | 60.3 (5.2) [15] |
| Beck Depression | 4.7 (5.9) [13] | 3.2 (5.0) [38] | 6.4 (7.6) [23] | 6.3 (5.6) [18] |
| PANAS Pos. | 35.9 (6.4) [13] | 26.0 (7.9) [39] | 28.7 (7.0) [23] | 28.2 (8.4) [17] |
| PANAS Neg, | 17.4 (7.9) [13] | 15.2 (4.0) [39] | 17.0 (6.7) [23] | 21.4 (6.6) [18] |
| Wellbeing WBQ | 211.0 (21.0) [13] | 188.4 (27.0) [37] | 188.1 (20.2) [23] | 176.2 (24.9) [18] |
| Num of Meds. | 0.3 (0.6) [14] | 1.7 (1.4) [40] | 0.5 (0.8) [23] | 2.5 (1.2) [22] |
| Work | 1.0 (0.2) [22] | 5.9 (1.0) [39] | 3.0 (1.6) [32] | 5.5 (1.5) [20] |
| Living | 1.1 (0.4) [22] | 2.2 (0.4) [45] | 1.9 (0.4) [32] | 2.5 (0.5) [24] |
| SEF Friends | 0.1 (0.4) [20] | 2.5 (0.7) [42] | 0.9 (0.8) [26] | 2.4 (0.7) [18] |
| Daily Living | 90.6 (12.7) [22] | 43.7 (16.6) [45] | 74.8 (11.9) [32] | 39.5 (18.2) [24] |

**Figure S3.** Classes identified from profiles omitting verbal IQ, non-verbal IQ and Comparative Severity Score.

**Figure S4.** Prediction of adult outcome classes for profiles omitting verbal IQ, non-verbal IQ and Comparative Severity Score.**Table S2.** Outcome profile classes when leaving out verbal IQ, non-verbal IQ and Comparative Severity Score from the profile of measures: means (standard deviations) [number of observations].

| Measure | Class 1  Best outcome | Class 2  Low-IQ ASD without behavioural problems | Class 3  High-IQ ASD with some behavioural and emotional problems | Class 4  Low-IQ ASD with behavioural problems |
| --- | --- | --- | --- | --- |
| Comp.Sev.Score | 3.9 (2.3) [29] | 6.3 (2.5) [48] | 5.5 (2.6) [17] | 6.1 (2.3) [24] |
| Verbal IQ | 104 (22.7) [30] | 39.6 (33.6) [49] | 96.3 (24.5) [18] | 25.8 (29.7) [26] |
| Non-verbal IQ | 102 (22.5) [30] | 45.4 (32.2) [49] | 96.7 (20.9) [18] | 32.2 (19.1) [26] |
| Hyperactivity | 1.6 (1.9) [26] | 5.8 (4.2) [47] | 5.7 (3.7) [10] | 19.9 (6.7) [21] |
| Irritability | 1.5 (2.6) [26] | 4.3 (3.7) [47] | 6.4 (4.3) [10] | 17.6 (7.0) [21] |
| CBCL total | 47.8 (7.7) [25] | 50.3 (8.3) [41] | 58.8 (7.2) [13] | 59.7 (5.0) [15] |
| Beck Depression | 2.6 (3.1) [21] | 2.9 (3.6) [45] | 17.7 (7.5) [9] | 5.6 (5.0) [17] |
| PANAS Pos. | 33.7 (7.0) [21] | 26.8 (7.6) [46] | 25.3 (8.5) [9] | 28.2 (8.6) [16] |
| PANAS Neg, | 14.3 (4.4) [21] | 15.1 (4.9) [46] | 27.1 (6.4) [9] | 20.9 (6.4) [17] |
| Wellbeing WBQ | 205.8 (21.9) [21] | 189.3 (25.2) [44] | 174.9 (17.5) [9] | 175.9 (25.6) [17] |
| Num of Meds. | 0.2 (0.5) [23] | 1.6 (1.3) [45] | 0.8 (1.4) [10] | 2.5 (1.2) [21] |
| Work | 1.4 (0.5) [30] | 5.7 (1.3) [44] | 2.7 (1.7) [18] | 5.6 (1.5) [21] |
| Living | 1.4 (0.5) [30] | 2.2 (0.4) [49] | 1.7 (0.5) [18] | 2.5 (0.5) [26] |
| SEF Friends | 0.3 (0.5) [28] | 2.4 (0.8) [43] | 0.9 (1.0) [15] | 2.6 (0.6) [20] |
| Daily Living | 84.8 (12.6) [30] | 49.6 (18.6) [49] | 77.1 (16.9) [18] | 36.2 (17.3) [26] |
